# Combination 20 and 23-gauge transconjunctival vitrectomy: A new approach

**DOI:** 10.4103/0301-4738.57158

**Published:** 2009

**Authors:** Atul Kumar, Aashish Kakkar, Shveta Jindal, R Rajesh

**Affiliations:** Department of Vitreous Retina service, Dr. Rajendra Prasad Centre for Ophthalmic Sciences, AIIMS, New Delhi-11 00 29, India

**Keywords:** Combined approach, complex surgeries, 20-23-gauge vitrectomy

## Abstract

The advent of smaller gauge instrumentation allows for minimally invasive vitreoretinal surgery (MIVS) as compared to conventional pars plana vitrectomy. Sutureless posterior segment surgery has the advantages of faster wound healing, minimal surgical trauma, decreased convalescence period besides reduced postoperative astigmatism; however, slower gel removal and limited peripheral vitreous dissection are disadvantages with smaller gauge systems. We herein describe a new technique combining 23-gauge and 20-gauge vitrectomy to improve the effectiveness and outcomes of vitreoretinal surgery.

The 23-gauge (23G) sutureless transconjunctival vitrectomy system utilizes tunneled two-step sclerotomies through the use of angled micro-vitreoretinal blade (23G) followed by a trocar-cannula system (DORC, Zuidland, The Netherlands) which provides an improved self-sealing incision and a low incidence of postoperative hypotony.[1–3] Use of 25-gauge vitrectomy system has also been described which uses full-thickness single-step non-tunneled sclerotomies,[4] however, there have been reports of increased risks of postoperative hypotony, choroidal effusion and endophthalmitis.[5] The narrow gauge cutters interfere with gel removal which necessitates a higher vacuum when 23/25G vitrectomy is performed. These narrow-gauge instrument shafts also have reduced stiffness; the weight required to move a cutter tip 120 degrees is 130 g for a 20G probe, 35 g for, a 23G probe and 14 g for the 25G probe.[6]

To overcome these limitations of pure 23G vitrectomy and to expand the existing indications for surgery, we describe a series of nine posterior segment surgeries with the use of combined 20G and 23G systems, using a single 20G superior vitrector port and two 23G, namely one superior port for endo-illumination and one infero-temporal infusion port.

## Materials and Methods

A series of nine combined 20 and 23G pars plana vitrectomies were performed by a single surgeon between October and December 2007. The indications for surgery included epiretinal membrane (ERM) peeling, macular hole surgery, diabetic vitreous hemorrhage, and diabetic tractional retinal detachment.

Operative outcome variables included operating time (time interval between first instrument contacting the conjunctiva and removal of infusion cannula), vitrectomy time (time required for completion of vitrectomy after insertion of the vitrectomy probe) and volume of balanced salt solution (BSS) plus used. Postoperative variables included postoperative visual acuity, media clarity, intraocular pressure (IOP) status, previous surgeries, phakic status, and comfort level of the patient [graded on a level from 1-4] [Table 1].

**Table 1:** Details of surgery and BSS fluid used

Indications for surgery	Av. Operating Time (in Min)	Av. Vitrectomy Time (in Min)*	BSS fluid used**	Previous surgeries	Phakic Status	Comfort level 1-4***
Full thickness	40	35	100 ml	Nil	Phakic	4
Macular hole					
PDR^+ combined RD#	50	40	185 ml	Nil	Phakic	2
PDR with Macular TRD##	40	35	130 ml	Nil	Phakic	3
Epiretinal membrane	30	20	100 ml	+(IOL Surgery)	Pseudophakic	4
Vitreous Hemorrhage (Hge)	40	30	150 ml	Nil	Phakic	4
Lasered PDR with Vitreous Hge	40	30	170 ml	Nil	Phakic	4
PDR with Vitreous Hge	30	20	110 ml	Nil	Phakic	4
Full-thickness Macular hole	30	20	100 ml	+(IOL Surgery)	Pseudophakic	3
Healed choroiditis with ERM###	30	20	60 ml	Nil	Phakic	3

*** Comfort level Grades 1 to 4, based on pain, watering and suture irritation (4 is high level of comfort, while 1 is excessive degree of discomfort)

* Av = Average

** BSS = Balanced salt solution

^ PDR = Proliferative Diabetic Retinopathy

# RD = Retinal Detachment

## TRD = Tractional Retinal Detachment

### ERM = Epiretinal Membrane

**Figure 1:**
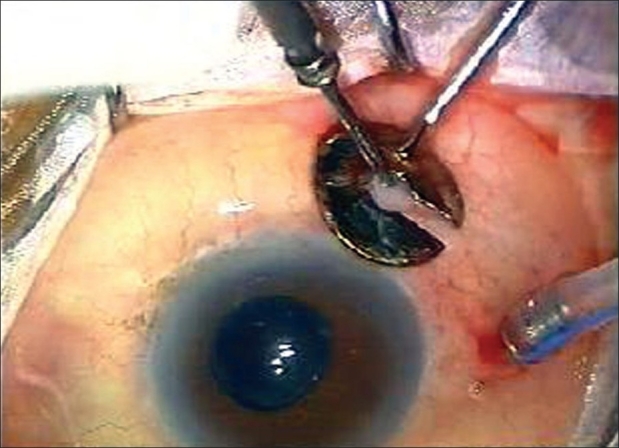
23G infusion sleeve in place (inferotemporal quadrant) while 23G trocar cannula being inserted in the superotemporal port

**Figure 2:**
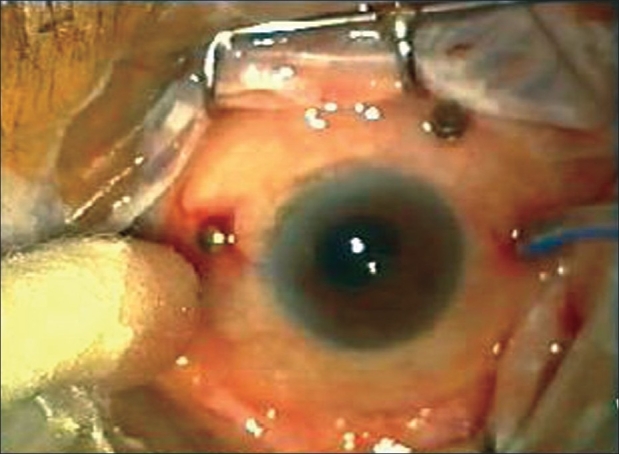
Two 23G openings, namely infusion port and superotemporal cannula visible, along with a single superonasal 20G port visible with a scleral plug in place

All patients received preoperative sedation and local anesthesia consisting of a peribulbar injection of 10 ml of a 50:50 mixture of 2% lidocaine and 0.15% bupivacaine. A 23G two-port setup for infusion and illumination (DORC, Zuidland, The Netherlands) was used along with a 20G vitrectomy through a third port. For the 23G opening, the conjunctiva was displaced 1-3 mm with a pressure plate with a central opening 3.5 mm from the edge. The 23G 45° angled microvitreoretinal blade (Bectin-Dickinson, USA) was inserted through the conjunctiva and sclera parallel and 3.5 mm posterior to the limbus through the central opening. A trocar-cannula was then inserted along the blade track while maintaining apposition of conjunctival and scleral opening with the pressure plate, the 23G infusion cannula was left inserted through the wound while the trocar was removed [Fig. 1]. Another similar port was made for the illumination probe. The third port included a localized peritomy and sclerotomy with a 20G microvitreoretinal blade [Fig. 2]. The hypotony using a combined 20-23G vitrectomy was best managed by increasing infusion bottle height to 50-60 cm and lowering suction settings to 75-100 mm Hg. Eyes being injected with silicon oil at the end of surgery had 1000cs silicone oil injected manually through the 23G infusion line. At the end of the surgery, 23G cannulae were removed and the site inspected for any wound leak which was sutured with a single stitch, only if found leaking, while the single 20G opening was sutured with 7-0 Vicryl.

## Results

**Figure 3:**
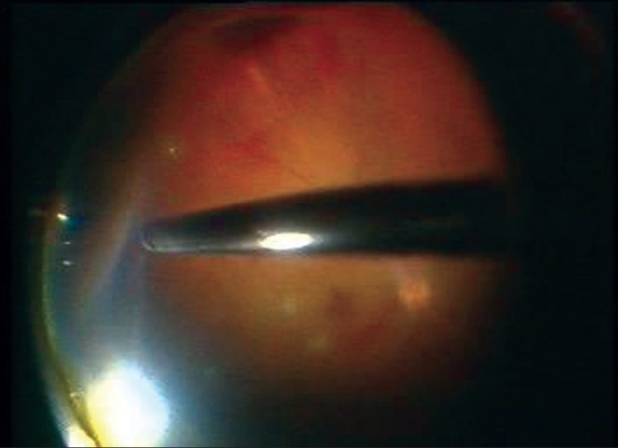
Combined (23+20G) vitrectomy in progress showing the 20G cutter effortlessly trimming the vitreous base in the periphery

The average operating time was 37 min with the average vitrectomy time being 27.77 min. The average BSS infusion fluid used was 122.77 ml [Fig. 3]. None of the cases required conversion to three-port 20G vitrectomy. Postoperatively six patients had a clear media with view of retina visible on indirect ophthalmoscopy (I/O) while one patient had a mild dispersed bleed; postoperative visual acuity on Day one ranged from 5/200 to 20/100. None of the eyes had postoperative hypotony. Two cases had 1000Cs viscosity silicon oil injection on completion of surgery. No case of endophthalmitis was observed during the immediate postoperative course [Table 1]. As it was a one-time evaluation of the patients, postoperative visual status was not followed up.

## Discussion

Sutureless self-sealing pars plana vitrectomy was first described by Chen in 1996.[1] Combined 20G and 23G vitrectomy, however, allows more thorough peripheral and more complex vitreoretinal maneuvers like near-complete vitreous base dissection, use of complex instruments in diabetic eyes for relieving traction, proliferative vitreoretinopathy (PVR) cases and eyes with intraocular foreign body (IOFB), eyes with dislocated lens and for silicon oil removal. The presence of two sutureless ports allows for low postoperative inflammation, good patient comfort, decreased conjunctival scarring and limited astigmatism.

Advantages of combination vitrectomy over a pure 23G vitrectomy are faster gel removal and enhanced ability to manipulate with a wide array of 20G instruments. Comparing 20G with 23G and 25G vitrectomy system has shown maximum cuts/min (CPM) of 2500 cuts/min for all three 20G, 23G and 25G cutters.[6] The tip of the cutter to the port distance is 0.009 inch for all gauge cutters. Fluidics show flow at 0 CPM to be 18cc/min for 20G cutter at the rate of 150 mm Hg and for 23G cutters at the rate of 450 mm Hg, while flow at peak cut rates (2500 CPM) for both 20G and 23G cutters is similar, 6cc/min and 7cc/min respectively. Hence 20G cutters are comparable to 23G cutters in terms of fluidics, port distance and flow rates. But the 20G cutter has distinctly stiffer shaft than a 23G cutter enabling easier peripheral vitreous dissection. However, there are certain situations during vitrectomy where interchanging of hands is required and it may be needed to enlarge the second superior port. This can be one difficulty in the combination 20 and 23G vitrectomy.

Thus with combined 20G with 23G vitrectomy[7–8], the indications for vitrectomy can be vastly expanded besides making it economically viable for the surgeon as he need not duplicate all 20G instruments to 23G. Hence it can be used as an alternative combination modality to the existing vitrectomy systems.

## References

[1] Chen JC (1996). Sutureless pars plana vitrectomy through self- sealing sclerotomies. Arch Ophthalmol.

[2] Eckardt C (2005). Transconjuctival sutureless 23 gauge vitrectomy. Retina.

[3] Fine HF, Iranmanesh R, Iturralde D, Spaide RF (2007). outcomes of 77 consecutive cases of 23 gauge transconjuntial vitrectomy surgery for posterior segment. Ophthalmology.

[4] Shimada H, Nakashi Zuka H, Mori R, Mizutani Y (2005). Expanded indications for 25G tranconjunctival vitrectomy. Jap J Ophthalmol.

[5] Scott IU, Flynn HW, Dev S, Saad S, Mittra RA, Arevalo JF (2008). Endophthalmitis after 25 Gauge and 20 Gauge pars plana vitrectomy. Retina.

[6] Charles S (2004). An engineering approach to vitreoretinal surgery continuing medical education. Retina.

[7] Hubschman JP, Gonzales CR, Bourla DH, Gupta A, Schwartz SD (2007). Combined 25- and 23-gauge surgery. a new sutureless vitrectomy technique. Ophthalmic Surg Lasers Imaging.

[8] Warrier SK, Jain R, Gilhotra JS, Newland HS (2008). Sutureless vitrectomy. Indian J Ophthalmol.

